# A Strength Endurance Exercise Paradigm Mitigates Deficits in Hypoglossal-Tongue Axis Function, Strength, and Structure in a Rodent Model of Hypoglossal Motor Neuron Degeneration

**DOI:** 10.3389/fnins.2022.869592

**Published:** 2022-06-30

**Authors:** Erika R. Murphy, Rebecca Thompson, Kate L. Osman, Chandler Haxton, Margaret Brothers, Li Lee, Kristen Warncke, Catherine L. Smith, Amy N. Keilholz, Ali Hamad, Mojgan Golzy, Filiz Bunyak, Lixin Ma, Nicole L. Nichols, Teresa E. Lever

**Affiliations:** ^1^Department of Speech, Language and Hearing Sciences, School of Health Professions, University of Missouri, Columbia, MO, United States; ^2^Department of Otolaryngology-Head and Neck Surgery, School of Medicine, University of Missouri, Columbia, MO, United States; ^3^Department of Biomedical Sciences, College of Veterinary Medicine, University of Missouri, Columbia, MO, United States; ^4^Department of Radiology, School of Medicine, University of Missouri, Columbia, MO, United States; ^5^Research Division, Biomolecular Imaging Center, Harry S. Truman Memorial Veterans’ Hospital, Columbia, MO, United States; ^6^Department of Electrical Engineering and Computer Science, University of Missouri, Columbia, MO, United States; ^7^Biostatistics Unit, Department of Family and Community Medicine, University of Missouri, Columbia, MO, United States; ^8^Dalton Cardiovascular Research Center, University of Missouri, Columbia, MO, United States

**Keywords:** motor neuron disease (MND), hypoglossal, tongue, dysphagia, exercise, rodent model

## Abstract

The tongue plays a crucial role in the swallowing process, and impairment can lead to dysphagia, particularly in motor neuron diseases (MNDs) resulting in hypoglossal-tongue axis degeneration (e.g., amyotrophic lateral sclerosis and progressive bulbar palsy). This study utilized our previously established inducible rodent model of dysphagia due to targeted degeneration of the hypoglossal-tongue axis. This model was created by injecting cholera toxin B conjugated to saporin (CTB-SAP) into the genioglossus muscle of the tongue base for retrograde transport to the hypoglossal (XII) nucleus via the hypoglossal nerve, which provides the sole motor control of the tongue. Our goal was to investigate the effect of high-repetition/low-resistance tongue exercise on tongue function, strength, and structure in four groups of male rats: (1) control + sham exercise (*n* = 13); (2) control + exercise (*n* = 10); (3) CTB-SAP + sham exercise (*n* = 13); and (4) CTB-SAP + exercise (*n* = 12). For each group, a custom spout with adjustable lick force requirement for fluid access was placed in the home cage overnight on days 4 and 6 post-tongue injection. For the two sham exercise groups, the lick force requirement was negligible. For the two exercise groups, the lick force requirement was set to ∼40% greater than the maximum voluntary lick force for individual rats. Following exercise exposure, we evaluated the effect on hypoglossal-tongue axis function (*via* videofluoroscopy), strength (*via* force-lickometer), and structure [*via* Magnetic Resonance Imaging (MRI) of the brainstem and tongue in a subset of rats]. Results showed that sham-exercised CTB-SAP rats had significant deficits in lick rate, swallow timing, and lick force. In exercised CTB-SAP rats, lick rate and lick force were preserved; however, swallow timing deficits persisted. MRI revealed corresponding degenerative changes in the hypoglossal-tongue axis that were mitigated by tongue exercise. These collective findings suggest that high-repetition/low-resistance tongue exercise in our model is a safe and effective treatment to prevent/diminish signs of hypoglossal-tongue axis degeneration. The next step is to leverage our rat model to optimize exercise dosing parameters and investigate corresponding treatment mechanisms of action for future translation to MND clinical trials.

## Introduction

Tongue weakness and atrophy are pervasive symptoms of motor neuron diseases (MNDs) (Brooks et al., 2000; Bruijn et al., 2004; Gonzalez de Aguilar et al., 2007; Mitchell and Borasio, 2007; Ravits et al., 2013; Turner et al., 2013; Waito et al., 2017) particularly amyotrophic lateral sclerosis (ALS), spinobulbar muscular atrophy/Kennedy’s disease, and progressive bulbar palsy (Baumer et al., 2014; Tiryaki and Horak, 2014; National Institute of Neurological Disorders and Stroke [NIH], 2019). For reasons that remain largely unknown, hypoglossal lower motor neurons (XII LMNs) innervating the tongue progressively degenerate in these patients, often resulting in life-threatening swallowing (dysphagia) and breathing (dyspnea) impairment (Hadjikoutis and Wiles, 2001; Corcia et al., 2008; Kurian et al., 2009; Kiernan et al., 2011). Treatments aimed at preventing XII LMN degeneration to preserve tongue function have not yet been identified, thus palliative/supportive intervention currently remains the standard of care. We propose that targeted tongue exercise may be a candidate treatment to significantly improve the quality and duration of life for MND patients.

Data on tongue exercise in MNDs are limited to only a few case studies (Dworkin and Hartman, 1979; Watts and Vanryckeghem, 2001) and animal model investigations (Ma et al., 2017) with variable findings, providing insufficient evidence to conclude whether tongue exercise is beneficial or harmful to MND patients (Plowman, 2015; Sheikh and Vissing, 2019). However, research outside the MND field has shown that tongue exercise improves upper airway/swallowing deficits caused by stroke (Robbins et al., 2007; Cullins et al., 2019), traumatic brain injury (Steele et al., 2013), Parkinson’s disease (Argolo et al., 2013; Ciucci et al., 2013; Wang et al., 2018), and biological aging (Connor et al., 2009; Kletzien et al., 2013) via putative neuroplastic mechanisms that are not yet well understood. Moreover, a growing body of evidence has emerged over the past two decades in favor of exercise training in general (i.e., not tongue-specific) in MND patients (Sheikh and Vissing, 2019). However, the optimal dose of time, intensity, and duration of exercise therapy remains to be identified for different MNDs as well as the different clinical stages of disease progression (Sheikh and Vissing, 2019; Tsitkanou et al., 2019).

To facilitate research in this area, we recently developed an inducible rat model of dysphagia due to selective degeneration of XII LMNs in the hypoglossal nucleus of the brainstem medulla, which provides the sole motor innervation to the tongue via the hypoglossal nerve. This model was created by injecting cholera toxin B conjugated to saporin (CTB-SAP) into the genioglossus muscle in the tongue base for retrograde transport to XII LMNs. Upon entering XII LMN cell bodies, CTB-SAP dissociates, leaving SAP free to bind to ribosomes and consequently halt protein synthesis, resulting in apoptotic cell death (Llewellyn-Smith et al., 2000; Lujan et al., 2010). Our recent investigations with this model revealed that a single CTB-SAP injection into the midline genioglossus of adult rats resulted in ∼60% XII LMN cell death within 9 days (Lind et al., 2018), with corresponding “downstream” degenerative changes in the XII nerve (i.e., denervation atrophy) and genioglossus (i.e., myofiber atrophy) (Lind et al., 2021) as well as development of swallowing-related deficits (slower lick and swallow rates) (Lind et al., 2018). In contrast, when control rats were tongue-injected with unconjugated CTB and SAP (CTB + SAP), only CTB was uptaken by XII nerve terminals for retrograde transport to XII LMNs, without resultant neuromuscular degenerative changes and corresponding swallowing-related deficits (Lind et al., 2018, 2021). Thus, our inducible rat model selectively involves the hypoglossal-tongue axis and provides a unique platform for studying the effects of targeted tongue exercise on tongue-related function, strength, and structure.

Here, we leveraged our rat model of hypoglossal-tongue axis degeneration to test the hypothesis that targeted tongue exercise can beneficially alter the clinical deficits we previously observed in this model. For this study, we developed a customized force-lickometer system to measure spontaneous lick force in rats during unrestrained drinking, and a tongue exercise spout that permits individualized resistance training overnight in the rat’s home cage. We utilized a high-repetition/low-resistance (i.e., strength endurance) exercise paradigm designed for muscle growth (Anderson and Kearney, 1982), which consisted of two non-consecutive overnight sessions. Following the exercise program, we evaluated the effect of targeted tongue exercise on hypoglossal-tongue axis function (*via* videofluoroscopy), strength (*via* force-lickometer), and structure (*via* MRI of the brainstem and tongue). These non-invasive to minimally invasive diagnostic tests were chosen because of their translatability to human medicine. Specifically, we hypothesized that: (1) sham exercise-treated CTB-SAP rats would develop behavioral evidence of dysphagia (i.e., reduced lick and swallow rates as previously shown (Lind et al., 2018), in addition to longer lick-swallow ratios and longer pharyngeal transit times, PTT) and reduced tongue strength (i.e., reduced lick force during drinking); (2) these behavioral-based deficits in tongue/swallow function and strength would be prevented by tongue exercise in CTB-SAP rats; (3) sham exercise-treated CTB-SAP rats would have degenerative structural changes that correspond to neuronal loss in the hypoglossal nucleus (i.e., enlarged 4th ventricle), as well as changes in the tongue (i.e., hyperintensity indicative of muscle fiber inflammation and fat infiltration and increased tongue thickness and volume) compared to control groups; and (4) these structural deficits would be prevented by tongue exercise in CTB-SAP rats. Results from this study may provide novel insight into translationally relevant clinical indicators of XII LMN degeneration and response to treatment. Moreover, demonstrating a beneficial effect of tongue exercise in this model would support its ongoing use in research to develop and optimize translationally feasible therapeutic strategies for MNDs.

## Materials and Methods

### Animals

Forty-eight male Sprague Dawley rats (Envigo Colony 208; Indianapolis, IN, United States) between 3 and 4 months of age (335–416 g) were included in this study. Rats were pair-housed in standard vivarium conditions (ambient temperature 20–26°C, humidity 30–70%, and standard 12-h light cycle) with unlimited access to food pellets (Purina Lab Diet 5008) and filtered tap water (pH adjusted to 3.5), except during experimental testing (described below). Daily health monitoring and routine surveillance for common rodent illnesses were performed by veterinary staff. All experimental procedures were approved by our Institutional Animal Care and Use Committee and conducted in accordance with the Guide for the Care and Use of Laboratory Animals within our USDA-licensed and AAALAC-accredited academic institution.

### Experimental Procedures

**FIGURE 1:**
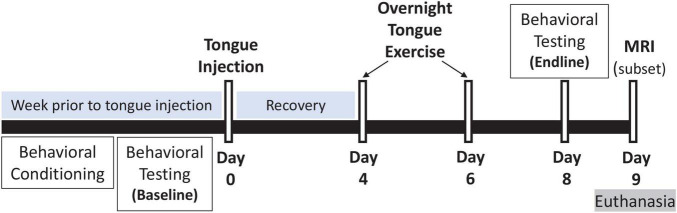
Experimental timeline. Our inducible rat model of targeted hypoglossal motor neuron degeneration has a 9-day timeline. On Day 0, rats received a single tongue injection of either control (unconjugated CTB + SAP) or conjugated CTB-SAP solution into the midline genioglossus. The week preceding tongue injection, rats underwent behavioral conditioning and baseline behavioral testing: (1) videofluoroscopic study (VFSS) to assess tongue motility and swallow function, and (2) force-lickometer testing to determine each rat’s maximum voluntary lick force (MVLF) during drinking. At Days 4 and 6, rats underwent an overnight (12-h) tongue exercise program consisting of low intensity (50% > MVLF) or sham (<4 g) exercise. Endline behavioral testing occurred on Day 8, followed by MRI (on a subset of 16 rats) and euthanasia on Day 9 (i.e., study endpoint).

Rats were randomly allocated to 4 experimental groups to study the effects of tongue resistance exercise on tongue-related function, strength, and structure, as summarized in Table 1. All rats received an intralingual injection of either unconjugated CTB + SAP (i.e., control) or conjugated CTB-SAP, followed by exposure to either tongue exercise (i.e., treatment) or sham exercise (i.e., sham treatment), as described in detail below. Before tongue injections and after exercise/sham exercise exposure, rats underwent behavioral testing of tongue function (*via* videofluoroscopy) and tongue strength (*via* force-lickometer). At the study endpoint (i.e., 9 days after tongue injection), a subset of 16 rats (1) control + sham exercise (*n* = 5); (2) control + exercise (*n* = 2); (3) CTB-SAP + sham exercise (*n* = 6); and (4) CTB-SAP + exercise (*n* = 3) underwent MRI of the brainstem and tongue to investigate corresponding structural changes that may correlate with behavioral findings. This study endpoint is the same as our previous studies (Lind et al., 2018, 2021), which was determined based on pilot data showing that other time points either did not result in dysphagia (4 days post tongue injection) or resulted in severe dysphagia (11–14 days post tongue injection). A summary of the 9-day experimental timeline is shown in Figure 1. All rats were euthanized on Day 9 using approved methods.

**TABLE 1:** Experimental group assignment.

Experimental groups		Group name	Sample size
Control	Sham Exercise	‘Control + Sham Exercise’	13
	Exercise	‘Control + Exercise’	10
CTB-SAP	Sham Exercise	‘CTB-SAP + Sham Exercise’	13
	Exercise	‘CTB-SAP + Exercise’	12
**Total**			**48**

*Sham Exercise = negligible (<4 g) lick force requirement; Exercise target = 50% greater than maximum voluntary lick force (MVLF).*

#### Intralingual Injections to Create an Inducible Rat Model of Targeted Hypoglossal Motor Neuron Degeneration

Our rat model was created as previously described (Lind et al., 2018, 2021). In brief, rats were anesthetized via 5% isoflurane in an induction chamber and immobilized in ear bars in the supine position on a custom-built tilt table to stabilize the head during tongue injections. For the remainder of the procedure, isoflurane (2–3%) was delivered via nose cone to extinguish hindlimb and jaw reflexes. The jaw was gently held open by a custom-built weighted pulley-mechanism looped around the mandibular incisors for unobstructed access to the tongue. Under light guidance (LED Stereotaxic Light, #59290, Stoelting; Wood Dale, IL, United States), fine forceps were used to gently lift the tongue for visualization of the frenulum, which was the anatomical landmark for targeted injection into the midline genioglossus muscle in the tongue base. Each rat received a single “control” injection (20 μg CTB + 25 μg SAP; unconjugated CTB + SAP) or CTB-SAP injection (25 μg of CTB conjugated to SAP) using a 50 μL Luer tip syringe (Microliter #705, Hamilton; Reno, NV, United States) and 26-gauge Luer lock needle (26G3/8, Becton, Dickinson and Company; Franklin Lakes, NJ, United States). The needle was angled at 45-degrees during insertion into the midpoint of the frenulum, with half of the bolus delivered at ∼8 mm depth (i.e., near maximum needle insertion) and the remainder at ∼4 mm (i.e., half the needle insertion depth) during needle retraction. All injections were performed by the same investigator for replicability of results. Rats were recovered from anesthesia and closely monitored for several hours to ensure resumption of food and water intake before being returned to standard vivarium conditions and daily health monitoring.

#### Behavioral Assessments of Tongue Motility, Swallowing Function, and Tongue Strength

Behavioral assessments included videofluoroscopic swallow study (VFSS) to assess tongue motility and swallowing function, and force-lickometer testing to assess tongue strength. Testing occurred at baseline (i.e., prior to tongue injection) and Day 8 (i.e., endline behavioral test point after exercise/sham exercise treatment). An extensive behavioral conditioning program was performed prior to baseline testing to ensure optimal performance during both tests at both timepoints. The behavioral conditioning and corresponding test protocols are described in detail below.

##### Behavioral Conditioning in Preparation for Behavioral Testing

All rats underwent 4 consecutive days of behavioral conditioning prior to baseline testing to ensure optimal performance on test days. For the first 2 days of conditioning, rats were exposed to the VFSS test solution (described below, but without radiographic contrast added) via standard vivarium bottles and a custom polycarbonate test chamber (with both end-caps removed for unimpeded pass-through exploration) for 2 h in the home cage. The final 2 days of conditioning took place in the actual test environment. For VFSS acclimation, rats were individually enclosed in a custom VFSS test chamber (described below) and placed on the remote-controlled platform within our miniature fluoroscope (described below). The remote-controlled platform was moved up/down and forward/backward for ∼10 min to simulate test conditions, without turning on the X-ray beam. Following VFSS acclimation, rats were individually contained in a custom force-lickometer chamber (described below) placed within the force-lickometer system (described below) for ∼10 min. During this time, rats had free access to the test solution (described below) from the force-lickometer spout (described below). Rats with drinking bouts of > 20 s (i.e., continuous drinking from the spout) were considered adequately acclimated for force-lickometer testing. Rats not meeting this criterion were returned to the home cage for up to one hour before undergoing a 2nd 10-min trial. Following each day of behavioral conditioning, rats were returned to the vivarium in the home cage.

##### Videofluoroscopic Swallow Study Testing

**FIGURE 2:**
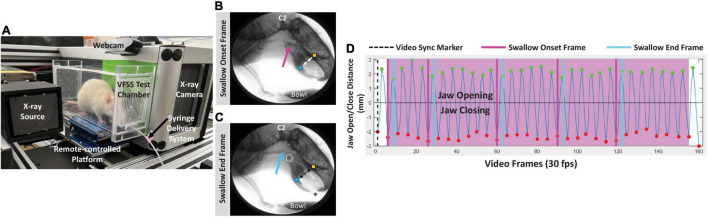
VFSS methods. **(A)** A rat undergoing VFSS testing in our miniature c-arm designed for use with rodents, with labeled components. **(B,C)** Representative lateral view radiographic images depicting the swallow onset frame **(B)** and swallow end frame **(C)** during VFSS testing. Note the bolus filling the vallecular space at swallow onset (pink arrow), and the bolus in the proximal esophagus (blue arrow) at the swallow end frame. The yellow and blue square markers on the upper and lower jaw, respectively, automatically tracked jaw open/close motion via our JawTrack™ software. Image contrast was adjusted to accentuate the bolus rather than soft tissue; the asterisk indicates the location of the tongue protruding toward the bowl (filled with liquid contrast agent) during drinking. C2 = 2nd cervical vertebra; white open circle = hyoid bone. The cross in the upper right quadrant is a 1 cm calibration marker. **(D)** Representative graph generated by our JawTrack™ software showing 5 s (@ 30 frames per second, fps) of uninterrupted licking based on jaw motion tracking **(B,C)**. The positive peaks (green dots) indicate maximum jaw opening/gape, whereas the negative peaks (red dots) indicate maximum jaw closure; the time stamp of each positive peak was used to calculate lick rate (Hz). Manually added event markers superimposed on the jaw motion graph indicate each pharyngeal swallow onset frame (pink lines) and pharyngeal swallow end frame (blue lines), which were used to calculate swallow rate (Hz) and pharyngeal transit time (PTT, ms). The black dashed line (i.e., video sync marker) moves in synchrony with the corresponding video frame when viewing video clips in our JawTrack™ interface.

Videofluoroscopic swallow study testing was performed as previously described (Lind et al., 2018). Briefly, rats underwent an overnight (12–16 h) water restriction prior to VFSS testing to motivate participation. During the water restriction period, a VFSS test chamber (clear polycarbonate, 25 cm long × 7.5 cm wide × 10 cm high) without end-caps was placed in the home cage for exploration. Only water was restricted; rats continued to have free access to food pellets. The following morning, rats were individually enclosed in the same home cage VFSS test chamber by attaching both end-caps; rats readily entered the test chamber when suspended by the tail over the chamber opening. The chamber (with enclosed rat) was positioned on the remote-controlled platform within our miniature low energy fluoroscope (The LabScope, Glenbrook Technologies; Randolph, NJ, United States), as shown in Figure 2A. With the continuous X-ray beam set to maximum power (40 kilovolts and 0.2 milliamperes), rats underwent videofluoroscopy in the lateral view while spontaneously drinking thin liquid contrast (by volume, 74.7% of 30% sucrose in DI water, 0.3% non-alcoholic vanilla extract, and 25% Omnipaque 350; GE Healthcare, Marlborough, MA, United States) from a peg-bowl inserted into an end-cap ∼2 mm above the chamber floor. The contrast solution was delivered to the peg-bowl using a custom syringe delivery system, which permitted undisturbed refilling (∼1.5 mL maximum volume) as needed between drinking bouts. The X-ray beam was turned on only when the rat was drinking from the bowl. Fluoroscopic videos were captured at 30 frames per second (fps) using video editing software (Pinnacle Studio 18, Pinnacle Systems, Inc.; Mountain View, CA, United States) on a desktop computer. Up to three 5-min trials were conducted per rat (spaced ∼15–30 min apart) until ∼2 min of drinking was recorded. Following testing, rats remained water restricted in preparation for force-lickometer testing (described below).

The fluoroscopic videos were analyzed in two steps. Step one entailed randomly selecting and splicing three non-overlapping 3–5-s clips of uninterrupted drinking from the peg-bowl using Pinnacle software. Step two entailed semi-automated analysis of jaw motion and bolus flow utilizing our custom jaw tracking software, JawTrack™ (see Supplementary Video 1), as previously described (Welby et al., 2020). Outcome measures included lick rate (i.e., number of licks per second; measured in cycles per second or Hertz, Hz) and swallow rate (i.e., number of swallows per second; measured in Hz), which we previously showed are impaired in our rat model (Lind et al., 2018). We also included two additional VFSS-measures for additional characterization of swallowing function: lick-swallow ratio (i.e., number of licks per swallow; a unitless measurement) and PTT (i.e., time between the swallow onset and swallow end frames; measured in milliseconds, ms). Quantification of these four outcome measures is explained in Figures 2B–D. The baseline and endline values were used to determine the exercise treatment effect on tongue motility and swallowing function.

##### Force-Lickometer Testing

**FIGURE 3:**
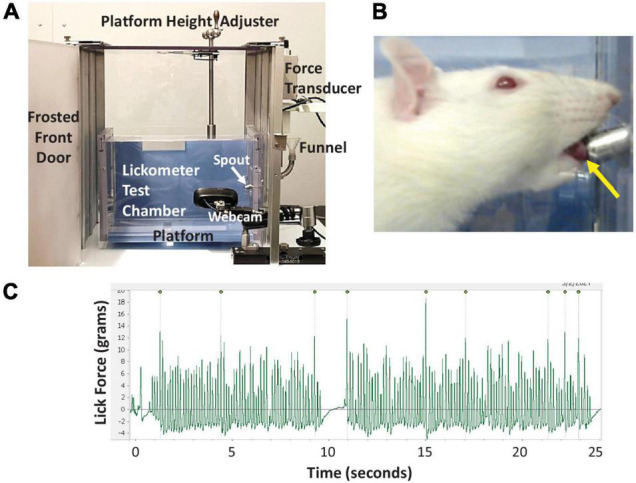
Lickometer methods. **(A)** Our custom force-lickometer system for use with rodents, with labeled components. During testing, the funnel is filled with a 30% sucrose solution, which rats drink from the spout. **(B)** Close-up image of a rat drinking from the spout, recorded via the lickometer webcam. Note the protruded tongue contacting the spout (yellow arrow). **(C)** Representative lick force graph generated by LabChart software showing 25 s of drinking. Peaks with the highest lick forces are automatically labeled; 9 peaks in this case. For this rat, each labeled peak is followed by 10–30 lower force licks (i.e., ∼1:20 ratio).

The maximum voluntary lick force (MVLF) of individual rats was measured using a modified force-lickometer system (Force Lickometer for Rat, Med Associates; Fairfax, VT, United States), as shown in Figure 3. Modifications included: (1) adding a site-built vertical lift platform for manual height positioning of individual rats at the lickometer spout, (2) substituting the pin-spout for a double ball-bearing spout that mimics our standard vivarium sipper tubes, and (3) substituting the data acquisition hardware and software with a PowerLab digitizer and LabChart software (ADInstruments; Colorado Springs, CO, United States) to permit graphic display of lick force data in synchrony with video recording of drinking behavior.

Rats underwent force-lickometer testing immediately following VFSS at each timepoint. During testing, rats were individually enclosed in a custom lickometer chamber (clear polycarbonate, 25 cm long × 8 cm wide × 15 cm high) positioned on a custom vertical lift platform within the lickometer system (Figure 3A). Rats readily entered the test chamber when suspended by the tail over the chamber opening. One end-cap has a centered vertical slip opening (5 cm) through which the lickometer spout is inserted to reach the chamber interior; this slit also accommodates raising/lowering of the lickometer platform for optimal height positioning of each rat during testing (Figure 3B). For each rat, the lickometer funnel reservoir was filled with 30% sucrose to motivate drinking from the spout. The spout was customized with a force-tension mechanism (described in the Tongue Exercise Paradigm subsection below) set to <4 g as a minimum force requirement during licking to obtain water access. This negligible force requirement was essential to prevent the spout from leaking during testing; however, it was well below the typical lick force of rats and thus did not prevent water access. Tongue contact against the spout was measured in grams (g) by the lickometer’s force transducer and digitized (PowerLab 8/30, ADInstruments) for recording and real-time graphic display (LabChart version 8, ADInstruments) on a dedicated computer. A webcam (V-u0018, Logitech; Newark, CA, United States) positioned within the lickometer system (but outside the test chamber) permitted synchronous video recording of drinking behaviors and lick force data via LabChart (Figures 3A–C). Up to three 5-min trials were conducted per rat (spaced ∼15-30 min apart) until ∼2 min of drinking was recorded. Following testing, rats were returned to the home cage with free access to food and water.

Calculation of MVLF for each rat began by identifying the 3–5 longest drinking bouts lasting at least 15 s in the LabChart files. Only lick-force data verified as actual licking at the spout in the synchronized videos were included in data analysis. Additionally, the initial ∼0.5 s of each bout (∼3–5 licks) was excluded to control for confounding extraneous head/body motion as the rat approached the spout to begin licking, which may artificially inflate the lick force values. From the identified drinking bouts, the peak-to-peak amplitude (g) of individual licks was automatically detected via LabChart (Figure 3C and Supplementary Video 2) and the corresponding data exported into a Microsoft Excel file. The 10 highest values across the multiple bouts were averaged to obtain each rat’s MVLF (Connor et al., 2009; Behan et al., 2012; Krekeler et al., 2020; Glass et al., 2021). As such, this approach is similar to measuring peak plantar pressure while walking at a natural, self-selected speed during kinematic gait analysis (Hessert et al., 2005; Nandikolla et al., 2017; Jasiewicz et al., 2022). The baseline MVLF value was used to determine the individualized exercise program for each rat (described below). MVLF values also were used to determine the exercise treatment effect.

#### Tongue Exercise Paradigm

**FIGURE 4:**
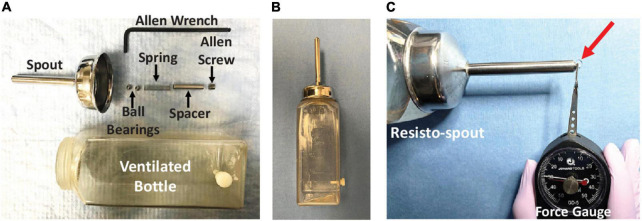
Resisto-spout design and calibration. **(A)** Our custom exercise spout (i.e., resisto-spout) with disassembled components (labeled), designed for home-cage use by individual rats. **(B)** The assembled resisto-spout replaces the standard vivarium water bottle during the overnight (12 h) exercise period. **(C)** Demonstration of lick force calibration (in grams) using a hand-held analog tension force meter (gauge). The spout force setting is manually adjusted to the target level for an individual rat by turning the Allen screw further in/out of the spout shaft, followed by re-testing with the force gauge. To measure the resisto-spout force setting, the resisto-spout and force gauge are secured in each hand and positioned perpendicular on an immovable tabletop. The tip of the gauge lever is precisely positioned to contact only the ball bearing that slightly protrudes from the spout tip. Pressure is incrementally applied to the ball bearing by manually moving the gauge dial slowly toward the resisto-spout in a single smooth, uninterrupted manner. The “reading” on the dial at which liquid begins to leak from the spout tip (red arrow) corresponds with the resisto-spout force setting.

Rats participated in a strength endurance tongue exercise paradigm on Day 4 and Day 6 post-tongue injection (see Figure 1 timeline), which necessitated single housing for the remainder of the study to ensure a personalized medicine approach. On both days, a custom exercise spout (i.e., resisto-spout, Figure 4) containing 30% sucrose solution was placed in the home cage of individually housed rats for 12 h overnight (i.e., ∼8:00 PM – 8:00 AM), coinciding with peak activity in these nocturnal rodents. The spout was customized with a manually adjustable tension spring mechanism (Figure 4A) with a force range of ∼2–50 g to accommodate sham exercise (<4 g) and exercise (∼20–35 g, based on unpublished pilot testing) conditions. Each resisto-spout force setting was calibrated immediately prior to use with one of two analog tension force meters (Figure 4C): low range (0–10 g; model GD-1, Jonard Tools, Elmsford, NY, United States) for sham exercise or high range (0–50 g; model GD-5, Jonard Tools) for exercise.

For rats in the two exercise groups (i.e., ‘control + exercise’ and ‘CTB-SAP + exercise’), the resisto-spout was set to 50% greater than baseline MVLF (i.e., 50% > MVLF) throughout the overnight exercise period. For example, a rat with a MVLF of 20 g would have the resisto-spout set to 30 g during exercise. Pilot testing (unpublished) revealed that CTB-SAP-injected rats were readily able to overcome this added low-force requirement to access the sucrose solution. Moreover, this 50% > MVLF target is well below the reported 100-150% increase in tongue force (during voluntary drinking) achieved by rats following an 8-week tongue resistance exercise program (Ma et al., 2017; Cullins et al., 2019; Glass et al., 2021). An important distinction here is that our approach is based on percent effort *above natural drinking behavior*, whereas most other research groups utilized force lickometer systems that permit estimation of maximum lick force capability (Connor et al., 2009; Behan et al., 2012; Cullins et al., 2019; Krekeler et al., 2020; Glass et al., 2021), thereby allowing the exercise target intensity level to be set to a percentage *below maximum effort* (i.e*., 50–80% of maximum effort)*, similar to the maximum bench press test to estimate training intensity percentages for weight-lifting (Grgic et al., 2020). Here, we were interested in the effect of low intensity exercise; therefore, as a starting point, we chose a relatively low effort requirement above MVLF (i.e., 50% > MVLF) as our target for low intensity tongue resistance exercise. Resisto-spouts for rats in the two sham exercise groups (i.e., ‘control + sham exercise’ and ‘CTB-SAP + sham exercise’) were set to <4 g, consistent with the negligible force setting used during force-lickometer testing. Resisto-spout force settings for all four groups were recorded for comparisons with and between groups.

Rats had free access to standard food pellets and enrichment materials during exercise/sham exercise, thus, the only cage-level difference was the substitution of the resisto-spout bottle (containing 30% sucrose solution) for the standard vivarium water bottle. Overnight fluid intake (in grams) was measured for each exercise night to estimate the level of exercise participation (i.e., lick frequency). We hypothesized that overnight fluid intake would be similar within and between rats over time, thus any identified exercise treatment effects would be due to differences in exercise level and group assignment (control vs. CTB-SAP) rather than lick frequency.

#### Live Magnetic Resonance Imaging of the Brainstem and Tongue

*In vivo* MRI scans were performed on a subset of 20 rats on Day 9 to determine the absence or presence of degeneration in the brainstem and tongue using a 7 T Bruker AVANCE III BioSpec MRI scanner (Bruker BioSpin Inc., Billerica, MA, United States) and a four-element phased-array radiofrequency (RF) coil. Rats were anesthetized with 1.0–3.5% isoflurane in oxygen via a nose cone and placed in supine (for brainstem imaging) or prone (for tongue) position. A physiological monitoring and gating system (SA Instruments, Inc., Stony Brook, NY, United States) was used to monitor vital signs and for triggering respiratory-gated MRI scans. Body temperature was maintained at 36–37°C with warm air circulating in the magnet bore. T2-weighted (T2W) MRI coronal and axial brain scans and axial tongue scans were performed with fat-suppression using multi-slice RARE (rapid acquisition with relaxation enhancement) spin echo sequence with a *b*-value of 0 or smaller than 30 s/mm^2^. Diffusion-weighted (DW) MRI sagittal scans of the brainstem were acquired using standard spin-echo DW sequence with a *b*-value of 750–1200 s/mm^2^. Respiratory gating was used to trigger each scan. Other imaging parameters included: 15–30 slices, slice thickness = 0.8 mm, in-plane resolution of 70–140 μm, TR (reputation time) = 2 s, TE (echo time) = 35 ms, RARE factor = 2, and 2 averages. Image analysis and processing were performed in ParaVision 6.0.1 software (Bruker BioSpin Corporation). The volume (mm^3^) of the brain 4th ventricle was measured as the sum of the areas multiplied by the slice thickness on the brain DW and T2W sagittal images at Bregma position –0.8, 0.0, and 0.8 mm. Axial T2W MRI was utilized to capture the oral tongue volume (cm^3^), which consisted of the anterior (mobile) body from the apex of the tongue to the root of the lower incisor teeth. Tongue volume was measured as the sum of the areas multiplied by the slice thickness on the tongue T2W axial images. The thickness (mm) of the tongue body was measured as the distance from dorsal to ventral surface of the tongue on three T2W axial images at 4.0–5.6 mm from the anterior apex of the tongue and calculated with an average value for each rat. Similarly, the width (mm) of the tongue blade was measured as the distance from left to right lateral surface of the tongue on five images at 3.2–6.4 mm from the apex of the tongue and calculated with an average value for each rat. The width (mm) of the tongue root was measured on the axial T2W image at the level of the root of the lower incisor teeth. Segmentations were performed for quantification of the volume of the brain 4th ventricle and tongue volume, thickness, and width using Segment (Medviso AB, Lund, Sweden) or ImageJ.

#### Statistical Analysis

Investigators involved with data collection were blinded to experimental group assignment until the study databases were created in either IBM SPSS Statistics 24 or SAS/STAT^®^ 13.1 software, which were used to perform statistical analyses. Data outliers for each variable were identified and re-checked for accuracy, but not removed from the dataset. The following dependent variables were assessed: (1) VFSS – lick rate (Hz), swallow rate (Hz), and PTT (ms); (2) force-lickometer – MVLF (g); (3) exercise intensity (g); (4) overnight fluid intake (g) during exercise; and (5) MRI – brain 4th ventricle volume (mm^3^), tongue volume (cm^3^), tongue thickness (mm), tongue blade width (mm), and tongue root width (mm). Averaged values for each animal were used for VFSS, force-lickometer, and MRI tongue (thickness and width) data, whereas raw/unaveraged data were used for fluid intake and MRI volume data (i.e., single value per animal per time point). Data for each variable were normally distributed (Shapiro–Wilk test, *p* > 0.05) and thus appropriate for parametric statistics. However, non-parametric statistics were used for MRI data to accommodate the small and unbalanced sample sizes (4–6 rats per group).

Resisto-spout force settings and corresponding exercise intensity were compared between groups using one-way analysis of variances (ANOVAs) and visualized using overlaid bloxplots and dot plots. Overnight fluid intake was compared between experimental groups using a mixed (group × time) repeated measures ANOVA (RMANOVA) and visualized using overlay plots (boxplots and dot plots). A separate Generalized Linear Regression Model (GLM) was fitted to assess the effect of exercise treatment on lick/swallow function and MVLF outcomes at Day 8 when controlling for baseline values. Interaction effects between baseline values and experimental groups were explored for each dependent variable to verify the identified treatment effects were not influenced by baseline values (i.e., no significant interactions). Bonferroni *post hoc* multiple comparison tests were performed to create 95% confidence intervals (CIs) and corresponding *p*-values for all pairwise differences between groups while controlling the familywise Type 1 error rate. The *R*^2^ scoring metric was used to measure the goodness of fit for the GLM models. Results were visualized via scatterplots with fitted regression lines from the GLM models. Additionally, overlay plots (boxplots and dot plots) were used to illustrate and summarize the descriptive statistics of lick/swallow function and MVLF outcomes at Day 8, with asterisks denoting the significant GLM findings. For MRI data, the non-parametric Kruskal–Wallis test (one-way ANOVA on ranks) was used to explore statistically significant differences between experimental groups for each outcome measure, followed by *post hoc* Dwass-Steel-Critchlow-Fligner multiple comparisons based on pairwise rankings. For all statistical tests, the significance threshold was set at α = 0.05.

## Results

All rats tolerated the experimental procedures without developing signs of stress or morbidity. As is expected with behavioral experiments, missing data occurred for each dependent variable for a variety of reasons (e.g., behavioral non-compliance, leaky resisto-spouts, technical difficulties, etc.). However, the group sample sizes for each behavioral test remained between 7 and 12 rats (summarized in each subsection below), which our prior studies have demonstrated is sufficient to detect statistical differences between groups (Lind et al., 2018).

### Tongue Exercise Paradigm

Meaningful exploration of potential treatment effects warrants full transparency of the rats’ participation in the treatment paradigm in order to draw appropriate conclusions. Thus, we have summarized essential details and data below.

#### Baseline Maximum Voluntary Lick Force and Corresponding Exercise Intensity Level

**FIGURE 5:**
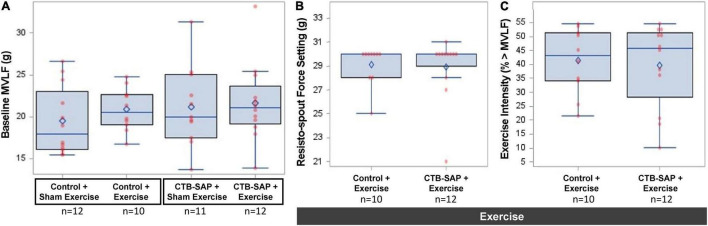
Baseline MVLF and corresponding resisto-spout force settings and exercise intensity. Baseline MVLF was used to determine the resisto-spout force setting and corresponding exercise intensity level for individual rats. **(A)** Baseline MVLF performance was similar (∼20 g) across the four experimental groups (i.e., no significant pairwise differences). **(B)** Resisto-spout force settings also were similar (∼29 g) between the two exercise groups. **(C)** The average exercise intensity was similar (∼40%) between both groups of exercised rats, which is consistent with our targeted low (<50%) intensity exercise paradigm. Error bars = standard error of the mean; *n* = group sample size; numbers in bars = average group value for each dependent variable.

Rats with inconclusive baseline MVLF values (*n* = 3) were allocated to sham exercise (i.e., ≤4 g force setting), leaving 45 rats for MVLF assessment. There was no significant difference in baseline MVLF between the four experimental groups (*F*_3,41_ = 0.605, *p* = 0.616), as shown in Figure 5A and summarized in Table 2. As expected (data not shown), there was a significant difference in exercise intensity between the four experimental groups (*F*_3,44_ = 1,017.71, *p* < 0.001), with *post hoc* comparisons revealing statistically significant differences between sham exercise and exercise groups (*p* < 0.001 for all comparisons) but not within each exercise group (*p* = 1.000 for all comparisons). As shown in Figure 5B and Table 2, the mean resisto-spout force setting was ∼29 g for both groups of exercise-treated rats, which corresponded to ∼40% > MVLF exercise intensity level (also shown in Figure 5C and Table 2).

**TABLE 2:** Descriptive statistics for baseline maximum voluntary lick force (MVLF) and corresponding resisto-spout force settings and exercise intensity by experimental group.

Experimental group	Sample size	Baseline MVLF (g)		Resisto-spout force setting (g)		Exercise intensity (% > MVLF)	
		Mean	SEM	Mean	SEM	Mean	SEM
‘Control + Sham Exercise’	12	19.5	1.18				
‘Control + Exercise’	10	20.9	0.82	29.05	0.54	40.1	3.62
‘CTB-SAP + Sham Exercise’	11	21.2	1.44				
‘CTB-SAP + Exercise’	12	21.7	1.37	28.92	0.783	38.5	4.27

*SEM, standard error of the mean. Exercise intensity (i.e., % > MVLF) is applicable only to the two exercise groups because the sham exercise groups had a negligible (i.e., <4 g) lick force requirement that was below their spontaneous lick force during voluntary drinking.*

Importantly, exercise intensity varied between rats in the two exercise groups (‘control + exercise’ and ‘CTB-SAP + exercise’), ranging from 10 to 53% higher than baseline MVLF values. While our initial plan was to use 50% > MVLF intensity for all rats, we elected to reduce the force requirement for rats that immediately began biting the spout at the onset of the overnight exercise period (i.e., 6 ‘control + exercise’ rats and 5 ‘CTB-SAP + exercise’ rats). For these cases, the threshold force requirement was reduced until stereotypical licking behavior resumed. Additionally, the analog technology of our resisto-spout allowed us to get close to, but typically a few grams away from, the target force setting; thus, some rats had slightly above or below the target 50% > MVLF value. Despite this variability, there was no significant difference in exercise intensity between the two groups (*F*_1,20_ = 0.078, *p* = 0.783). The mean (standard error) exercise intensity levels were 40.1% (3.62) for the ‘control + exercise’ group and 38.5% (4.27) for the ‘CTB-SAP + exercise’ group. Graphic display of the data distribution is shown in Figure 5C. As a reminder, the resisto-spout for the two sham-exercise groups (‘control + sham exercise’ and ‘CTB-SAP + sham exercise) was set to a negligible (<4 g) force requirement for fluid access, which was well below each rat’s MVLF and thus did not constitute exercise for these two groups.

#### Overnight Fluid Intake to Estimate Exercise Participation

Of the two exercise nights (Days 4 and 6), rats consumed more 30% sucrose solution on the 2nd night (i.e., main effect of time; *F*_1,31_ = 6.533, *p* = 0.016). However, there was no significant difference in overnight fluid intake between experimental groups (main effect, *F*_3,31_ = 0.320, *p* = 0.811), nor was there a time × group interaction (*F*_3,31_ = 0.316, *p* = 0.813). Thus, fluid intake (30% sucrose) was similar between groups at both exercise timepoints (Day 4 and Day 6). Prior to this study, we determined that naïve (non-injected, non-exercised) rats weighing ∼350 g consumed an average of 14 g water during a 12-h overnight period (unpublished), which is consistent with published values of 9–12 g water per 100 g rat per 24 h (Holdstock, 1973). The rats in this study drank an average of 29 g sucrose solution per night (Table 3), which equates to ∼32 mL (i.e., 1.1 g/mL 30% sucrose solution). Thus, the rats consumed ∼ 2 times more volume than normal each night, which demonstrates the added value of providing sucrose rather than plain water to motivate enhanced participation (i.e., higher lick frequency) during the exercise program. Moreover, using published data of 200 licks per mL sucrose solution in naïve rats (Hsiao and Fan, 1993), we estimated that our rats licked approximately 6,400 times (i.e., 32 mL average overnight intake X 200 licks per mL sucrose solution) per night. However, their stereotypical lick pattern consisted of intermittently higher lick forces to obtain water from the spout, followed by multiple lower lick forces to “manage” the dispensed water (i.e., ∼1:20 ratio, as shown in Figure 3). This equates to an estimated 320 “forceful” licks per rat (i.e., 6,400 ÷ 20) to access the sucrose solution during each 12-h exercise period, which is consistent with a high repetition exercise paradigm.

**TABLE 3:** Descriptive statistics for overnight fluid intake during exercise by experimental group and exercise time point (Days 4 and 6).

Experimental group	Sample size	Fluid intake (grams)					
		Day 4		Day 6		Average	
		Mean	SEM	Mean	SEM	Mean	SEM
‘Control + Sham Exercise’	7	29.3	4.4	33.7	3.5	31.5	3.4
‘Control + Exercise’	8	23.2	4.2	30.9	3.2	27.0	3.2
‘CTB-SAP + Sham Exercise’	9	27.3	3.9	30.6	3.1	29.0	3.0
‘CTB-SAP + Exercise’	11	28.2	3.5	31.5	2.8	29.9	2.7
**Grand total**						**29.3**	**3.1**

*SEM, standard error of the mean. Fluid = 30% sucrose in standard vivarium water (pH 3.5).*

### Tongue Exercise Treatment Effect on Behavioral Videofluoroscopic Swallow Study Measures of Lick/Swallow Function

**FIGURE 6:**
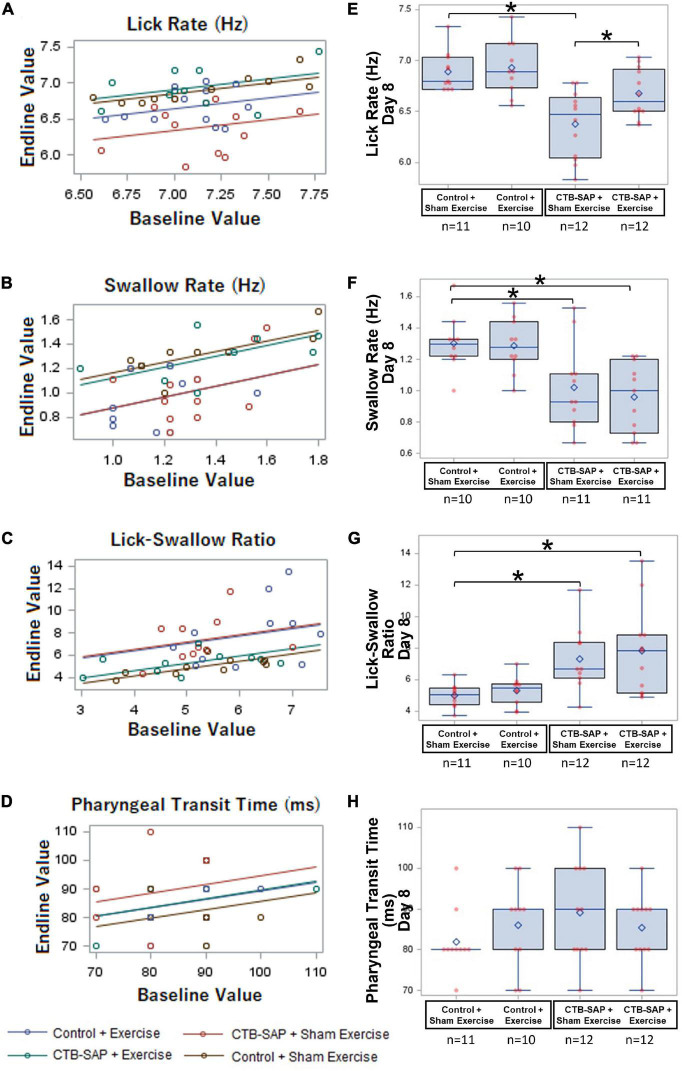
Effect of tongue exercise on VFSS-based lick and swallow function. **(A–D)** Scatter plots of baseline vs. endline values and the regression lines from the fitted generalized regression model are shown for each VFSS outcome measure. The estimated difference between any pair of groups is the vertical distance between the corresponding lines. Significant differences between the regression lines are summarized in **(E–H)** as corresponding overlay boxplots (median, quartiles, and whiskers; mean = diamond) and dot plots (individual data points) to highlight the significant treatment effects at the Day 8 time point in our CTB-SAP model. Data points outside the whiskers are considered mild outliers; there were no extreme outliers. Note that only 3 of the 4 VFSS outcome measures (i.e., lick rate, swallow rate, and lick-swallow-ratio) were significantly impaired in the ‘CTB-SAP + sham exercise’ group, but only lick rate was significantly improved in the ‘CTB-SAP + exercise’ group. Specifically, compared to the ‘control + sham exercise’ group, lick rate was significantly slower in the ‘CTB-SAP + sham exercise’ group (*p* < 0.001) but not the ‘CTB-SAP + exercise’ group (*p* = 0.0689). Moreover, lick rate was significantly different between the two CTB-SAP groups (i.e., faster for the ‘CTB-SAP + exercise’ group; *p* < 0.042). Thus, our CTB-SAP model develops impaired lick motility that is beneficially improved by targeted tongue exercise. **(B)** Swallow rate was significantly slower in both CTB-SAP groups (‘CTB-SAP + sham exercise’: *p* = 0.0017; ‘CTB-SAP + exercise’: *p* = 0.0021) compared to the ‘control + sham exercise’ group, but targeted tongue exercise had no effect on this VFSS outcome measure. **(C)** Lick-swallow ratio was significantly lower in both CTB-SAP groups (‘CTB-SAP + sham exercise’: *p* = 0.0048; ‘CTB-SAP + exercise’: *p* = 0.0072) compared to the ‘control + sham exercise’ group but was unaffected by tongue exercise. **(D)** Pharyngeal transit time was not significantly different between experimental groups. Error bars = standard error of the mean; *n* = group sample size; asterisks indicate significant difference (*p* < 0.05) between pairwise groups.

As summarized in Table 4, three of the four VFSS outcome measures were significantly different between the four experimental groups when controlling for baseline values: lick rate (*F*_4,40_ = 9.97, *p* < 0.001), swallow rate (*F*_4,37_ = 9.59, *p* < 0.0001), and inter-swallow interval (*F*_4,36_ = 6.28, *p* = 0.0006). The model for PTT was weak (*R*^2^ = 0.28) and not statistically significant (*F*_4,37_ = 1.29, *p* = 0.2924). Importantly, there was no significant interaction effect of baseline and experimental group for the VFSS outcome measures, thus confirming that any subsequently identified treatment effects via *post hoc* testing (summarized separately below for each VFSS outcome measure) were not influenced by baseline values. Figures 6A–D summarizes the GLM data for each VFSS outcome measure as regression plots with baseline versus endline values for individual animals within each experimental group. Figures 6E–H shows overlay plots summarizing the corresponding descriptive statistics for each outcome measure at Day 8 to highlight the identified treatment effects. Descriptive statistics for each VFSS outcome measure are presented in Table 5.

**TABLE 4:** GLM fitted model summary table for behavioral outcome measures at day 8 when controlling for baseline values.

Outcome measure	Parameter	Estimate	Standard	*t*-Value	Pr > |t|
			Error of Estimate		
Lick Rate (Hz) (*R*^2^ = 0.50) *p* < 0.0001	Intercept	4.796	0.910	5.27	<0.0001
	Lick Rate Baseline Value	0.293	0.127	2.31	0.0261
	Control + Exercise	0.058	0.111	0.52	0.6054
	CTB-SAP + Sham Exercise	–0.511	0.105	–4.85	**<0.0001**
	CTB-SAP + Exercise	–0.197	0.106	–1.87	0.0689
	Control + Sham Exercise	0	.	.	.
Swallow Rate (Hz) (*R*^2^ = 0.51) *p* < 0.0001	Intercept	0.722	0.184	3.93	0.0004
	Swallow Baseline Value	0.441	0.132	3.33	0.002
	Control + Exercise	–0.037	0.086	–0.44	0.6651
	CTB-SAP + Sham Exercise	–0.282	0.083	–3.39	**0.0017**
	CTB-SAP + Exercise	–0.283	0.085	–3.31	**0.0021**
	Control + Sham Exercise	0	.	.	.
Lick-Swallow Ratio (*R*^2^ = 0.41) *p* = 0.0006	Intercept	1.471	1.650	0.89	0.3785
	Lick-Swallow Ratio Baseline	0.656	0.289	2.27	0.0293
	Control + Exercise	0.514	0.807	0.64	0.5284
	CTB-SAP + Sham Exercise	2.413	0.803	3.01	**0.0048**
	CTB-SAP + Exercise	2.335	0.819	2.85	**0.0072**
	Control + Sham Exercise	0	.	.	.
PTT (ms) (*R*^2^ = 0.12) *p* = 0.2924	Intercept	56.027	17.118	3.27	0.0023
	PTT Baseline Value	0.299	0.194	1.54	0.1314
	Control + Exercise	3.701	4.376	0.85	0.4031
	CTB-SAP + Sham Exercise	8.638	4.388	1.97	0.0565
	CTB-SAP + Exercise	3.373	4.272	0.79	0.4348
	Control + Sham Exercise	0	.	.	.
MVLF (g) (*R*^2^ = 0.28) *p* = 0.0183	Intercept	18.602	3.338	5.57	<0.0001
	MVLF Baseline Value	0.073	0.164	0.44	0.6602
	Control + Exercise	0.094	1.598	0.06	0.9535
	CTB-SAP + Sham Exercise	–4.181	1.607	–2.6	**0.0135**
	CTB-SAP + Exercise	1.214	1.612	0.75	0.4565
	Control + Sham Exercise	0	.	.	.

***‘**Control + Sham Exercise’ was considered as the reference for experimental group pairwise comparisons for each outcome measure. Statistically significant pairwise comparisons are bolded and highlighted in yellow.*

**TABLE 5:** Descriptive statistics for behavioral outcome measures by experimental group at day 8.

Outcome Measures	CTB SAP + Exercise		CTB SAP + Sham Exercise		Control + Exercise		Control + Sham Exercise	
	Mean	SEM	Mean	SEM	Mean	SEM	Mean	SEM
Lick Rate (Hz)	6.67	0.07	6.38	0.1	6.93	0.09	6.89	0.06
Swallow Rate (Hz)	0.96	0.07	1.02	0.08	1.29	0.05	1.3	0.06
Lick Swallow Ratio	7.86	0.86	7.33	0.6	5.3	0.29	4.99	0.23
PTT (ms)	85.45	2.47	89.09	3.68	86	3.4	82	2.49
MVLF (g)	21.37	1.4	15.96	0.76	20.22	1.32	20	0.68

#### Tongue Exercise Mitigates Deficits in Lick Rate but Not Swallow Timing Measures in Cholera Toxin B Conjugated to Saporin-Injected Rats

##### Lick Rate

As hypothesized, lick rate was significantly slower in the ‘CTB-SAP + sham exercise’ group compared to the ‘control + sham exercise’ group [*d* = 0.51, 95% CI = (0.23, 0.80), *p* < 0.001], thus demonstrating that CTB-SAP causes impaired tongue motility congruent with our prior study (Lind et al., 2018). Also as hypothesized, lick rate in the ‘CTB-SAP + exercise’ group was not statistically different from the ‘control + sham exercise’ group [*d* = 0.21, 95% CI = (–0.065, 0.50), *p* = 0.0689] but was significantly faster than the ‘CTB-SAP + sham exercise’ group [*d* = 0.29, 95% CI = (0.02, 0.58) *p* < 0.042], thus providing evidence of a beneficial exercise treatment effect. Interestingly, the exercise intensity training was sufficiently low to result in no significant difference in lick rate between the two control groups: ‘control + sham exercise’ and ‘control + exercise’ [*d* = 0.25, 95% CI = (–0.035, 0.54), *p* = 0.6054]. These findings, summarized in Table 4 and Figure 6E, demonstrate that targeted tongue resistance exercise in CTB-SAP-injected rats mitigates tongue motility dysfunction.

##### Swallow Rate

As hypothesized, swallow rate was significantly slower in the ‘CTB-SAP + sham exercise’ group compared to ‘control + sham exercise’ rats [*d* = 0.28, 95% CI = (0.05, 0.50), *p* = 0.0017], thus demonstrating that CTB-SAP causes impaired swallow function congruent with our prior study (Lind et al., 2018). However, swallow rate also was significantly slower for the ‘CTB-SAP + exercise’ group compared to ‘control + sham exercise’ rats [*d* = 0.34, 95% CI = (0.11, 0.57), *p* < 0.0021], but there was no significant difference in swallow rate between the two CTB-SAP groups [*d* = 0.06, 95% CI = (–0.15, 0.28, *p* = 0.9969]. Thus, contrary to our hypothesis, tongue exercise had no apparent beneficial treatment effect on swallow rate in our model. Additionally, there was no significant difference in swallow rate between the two control groups: ‘control + sham exercise’ and ‘control + exercise’ [*d* = 0.014, 95% CI = (–0.22, 0.25), *p* = 0.6651]. These findings are summarized in Table 4 and Figure 6F.

##### Lick-Swallow Ratio

As hypothesized, lick-swallow ratio was significantly higher in the ‘CTB-SAP + sham exercise’ group compared to the ‘control + sham exercise’ group [*d* = 2.34, 95% CI = (0.18, 4.5), *p* = 0.0048], which provides a new outcome measure for assessing treatment effects in this rat model. However, lick-swallow ratio also was significantly higher for the ‘CTB-SAP + exercise’ group compared to ‘control + sham exercise’ rats [*d* = 0.34, 95% CI = (0.11, 0.57), *p* < 0.0072], but there was no significant difference in lick-swallow ratio between the two CTB-SAP groups [*d* = 0.52, 95% CI = (–1.58, 2.63), *p* = 0.9254] or the two control groups [*d* = 0.31, 95% CI = (–1.92, 2.55), *p* = 0.5284]. Thus, similar to the results of swallow rate, tongue exercise had no apparent beneficial treatment effect on lick-swallow ratio. These findings are summarized in Table 4 and Figure 6G.

##### Pharyngeal Transit Time

Although not significant, the ‘CTB-SAP + sham exercise’ group had the longest PTT. Moreover, the PTT distribution for the ‘CTB-SAP + exercise’ group was similar to the two control groups, suggesting a potential beneficial effect of tongue exercise may emerge with larger group sample sizes. These findings are summarized in Table 4 and Figure 6H.

### Lick Force Deficit During Voluntary Drinking in Cholera Toxin B Conjugated to Saporin-Injected Rats Is Mitigated by Targeted Strength Endurance Exercise

**FIGURE 7:**
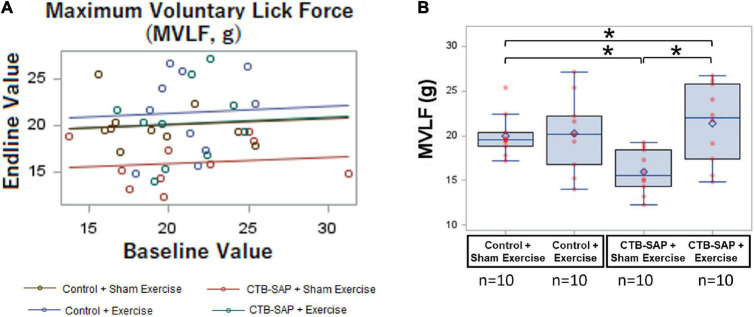
Effect of tongue exercise on maximum voluntary lick force (MVLF). MVLF data are graphically summarized as a GLM regression plot **(A)** and corresponding overlay boxplots and dot plots **(B)** highlighting treatment effects at Day 8. Data points outside the boxplot whiskers are considered mild outliers. Compared to the ‘control + sham exercise’ group, MVLF was significantly reduced in the ‘CTB-SAP + sham exercise’ group (*p* = 0.0135). Moreover, MVLF was significantly different between the two CTB-SAP groups (i.e., higher for the ‘CTB-SAP + exercise’ group; *p* = 0.0016). MVLF also was significantly higher for the ‘CTB-SAP + exercise’ group compared to the ‘control + sham exercise’ group (*p* = 0.027). Thus, our CTB-SAP model develops impaired tongue strength that is beneficially improved (i.e., surpasses control levels) by targeted tongue exercise. Error bars = standard error of the mean; *n* = group sample size; asterisk indicates a significant difference (*p* < 0.05) between pairwise groups.

As summarized in Table 4, MVLF was statistically different between the four experimental groups at Day 8 when controlling for baseline values (*F*_4,35_ = 3.42, *p* = 0.0183), as shown in Figure 7A. As hypothesized, the ‘CTB-SAP + sham exercise’ group performed significantly worse (i.e., had lower MVLF values) than the ‘control + sham exercise’ group [*d* = 5.405, 95% CI = (1.16, 9.65), *p* = 0.0135], thus providing novel evidence of a lick force deficit in this model. Also as hypothesized, MVLF was significantly higher in the ‘CTB-SAP + exercise’ group compared to the ‘CTB-SAP + sham exercise’ group [*d* = 5.405, 95% CI = (1.161, 9.649), *p* = 0.0016]. Interestingly, MVLF for the ‘CTB-SAP + exercise’ group was significantly higher than the ‘control + sham exercise’ group [*d* = 1.4, 95% CI = (–2.87, 5.6), *p* = 0.027] but was not significantly different from the ‘control + exercise’ group [*d* = 1.15, 95% CI = (–3.1, 5.39), *p* = 0.4818]. Moreover, there was no significant difference between the two control groups: ‘control + sham exercise’ and ‘control + exercise’ [*d* = 0.218, 95% CI = (–4.03, 4.46), *p* = 0.9535]. Collectively, these results demonstrate that our CTB-SAP rat model develops impaired tongue strength (i.e., lick force), and a strength-endurance tongue exercise paradigm preserves tongue strength at/above control levels. Figure 7B graphically summarizes these findings, and descriptive statistics are presented in Table 5.

### Tongue Exercise Mitigates Structural Degeneration in the Brainstem and Tongue of Cholera Toxin B Conjugated to Saporin-Injected Rats

Magnetic Resonance Imaging of the brainstem and tongue suggest further degenerative changes in the hypoglossal-tongue axis; however, statistical significance was reached only for some of the tongue but not brainstem MRI measures. Descriptive statistics for all MRI outcome measures are presented in Table 6.

**TABLE 6:** Descriptive statistics for MRI outcome measures by experimental group at day 9.

Outcome Measures	CTB SAP + Exercise		CTB SAP + Sham Exercise		Control + Exercise		Control + Sham Exercise	
	Mean	SEM	Mean	SEM	Mean	SEM	Mean	SEM
Tongue Volume (cm^3^)	0.495	0.008	0.543	0.014	0.489	0.012	0.488	0.015
Tongue thickness (mm)	5.123	0.117	5.791	0.237	5.097	0.302	4.815	0.112
Tongue width at root (mm)	7.233	0.231	7.404	0.275	7.693	0.115	7.086	0.231
Tongue width at blade (mm)	8.462	0.280	8.836	0.167	8.160	0.249	8.604	0.251
4th Ventricle volume (mm^3^)	3.893	0.402	5.052	0.337	3.431	0.393	3.729	0.279

**FIGURE 8:**
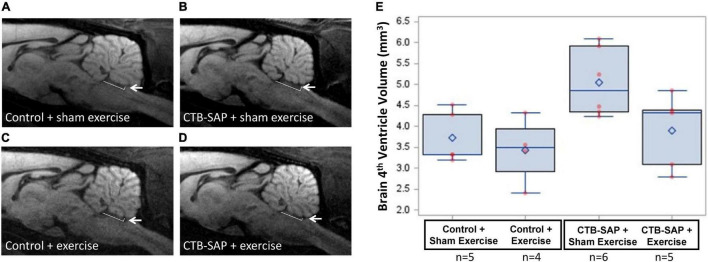
Structural changes in the brainstem in the absence and presence of tongue exercise. **(A–D)** *In vivo* diffusion weighted MRI of brainstem sagittal slice at Bregma position 0.0 mm shows a trend for an enlargement of the 4th ventricle in sham exercise-treated CTB-SAP rats (**B**, denoted by white arrow) vs. sham exercise-treated controls **(A)**, suggesting degeneration of neighboring brainstem tissue (i.e., XII nucleus). Following tongue exercise, the size of the 4th ventricle (denoted by white arrow) in CTB-SAP rats **(D)** appeared to resemble that of sham exercise-treated **(A)** and exercise-treated **(C)** control rats, suggesting that tongue exercise may prevent or decrease/slow degeneration in CTB-SAP rats and provides sufficient preliminary evidence to continue collecting these non-invasive translational measurements with larger group sample sizes. **(E)** MRI segmented 4th ventricle volume showed no significant differences across groups (*p* = 0.075). Error bars = standard error of the mean; *n* = group sample size.

For MRI of the brain, no significant differences in the 4th ventricle volume were identified across experimental groups (*p* = 0.075). However, as shown in Figure 8, 4th ventricle volume was notably higher in ‘CTB-SAP + sham exercise’ rats compared to the other three groups, suggesting significance may emerge with larger group sample sizes that permit application of more robust statistical analysis approaches.

**FIGURE 9:**
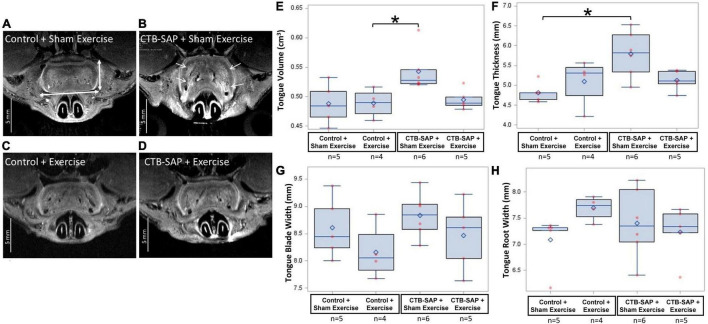
Structural changes in the tongue in the absence and presence of tongue exercise. **(A–D)** *In vivo* T2-weighted MRI of the axial images at the A-P position 4.8 mm from the anterior apex of the tongue shows hyperintensity (i.e., increased brightness, denoted by white arrows) and increased tongue thickness and volume in sham exercise-treated CTB-SAP rats **(B)** vs. sham exercise-treated controls **(A)**, suggesting hypertrophy of the tongue and is consistent with muscle fiber inflammation and fatty replacement of atrophied muscle fibers. Following tongue exercise, tongue thickness, volume, and intensity in CTB-SAP rats **(D)** resembled that of sham exercise-treated and exercise-treated control rats **(A,C)**, suggesting that tongue exercise may prevent or decrease/slow degenerative structural changes in CTB-SAP rats. Tongue thickness and width are indicated by vertical and horizontal double arrows, respectively in **(A)**. **(E)** MRI segmentation of tongue volume showed a significant increase in the ‘CTB-SAP + sham exercise’ group vs. the ‘control + exercise’ group (*p* = 0.05) but was not significantly different compared to ‘CTB SAP + exercise’ (*p* = 0.08) and ‘control + sham exercise’ (*p* = 0.18) groups. **(F)** Tongue thickness was significantly increased in the ‘CTB-SAP + sham exercise’ group vs. the ‘control + sham exercise group’ (*p* = 0.05). **(G,H)** The tongue blade and root widths were not significantly different across groups (*p* = 0.251 and *p* = 0.1805, respectively). Error bars = standard error of the mean; *n* = group sample size.

For MRI of the tongue, statistically significant differences were identified for tongue volume (*p* = 0.029) and thickness (*p* = 0.0395) but not for tongue width measured at either the blade (*p* = 0.251) or root (*p* = 0.1805) regions. As shown in Figure 9, tongue volume and thickness were notably greater in the ‘CTB-SAP + sham exercise’ group compared to the other three groups. Specifically, tongue volume was significantly greater in the ‘CTB-SAP + sham exercise’ group compared to ‘control + exercise’ rats (*p* = 0.05). Although not significant, tongue volume was greater in ‘CTB-SAP + sham exercise’ rats compared to ‘CTB-SAP + exercise’ (*p* = 0.08) and ‘control + sham exercise’ (*p* = 0.18) groups. Tongue thickness was significantly greater in ‘CTB-SAP + sham exercise’ compared to ‘control + sham exercise’ (*p* = 0.05) groups. We also qualitatively observed increased bilateral hyperintensity in the intrinsic muscles (i.e., verticalis and transversus muscles) of the anterior tongue in ‘CTB-SAP + sham exercise’ rats *vs*. controls. The diffuse-pattern of the hyperintensity is clearly visible and is indicative of muscle fiber inflammation and infiltrations of fatty replacement of atrophied muscle fibers in ‘CTB-SAP + sham exercise’ rats. Collectively, these preliminary results suggest hypertrophy of the tongue and a beneficial treatment effect of tongue exercise on tongue volume and thickness in CTB-SAP-injected rats.

## Discussion

The main findings from this study were that: (1) sham exercise-treated CTB-SAP rats had behavioral evidence of dysphagia (i.e., reduced lick and swallow rates as previously shown,(Lind et al., 2018) and longer lick-swallow ratios) and reduced tongue strength (i.e., lick force); (2) our strength endurance tongue exercise program resulted in preserved lick rate and lick force in CTB-SAP rats but had no effect on swallow timing deficits; (3) structural degeneration in the brainstem and tongue in sham exercise-treated CTB-SAP rats was observed via *in vivo* MRI; and (4) tongue exercise appears to mitigate this structural degeneration in CTB-SAP rats. These novel findings collectively suggest that a strength endurance exercise program targeting the tongue may be a feasible treatment for hypoglossal-tongue axis degeneration in motor neuron diseases such as ALS.

Importantly, we purposely chose to start the tongue exercise program on Day 4 based on published work showing it takes 3 days following intrapleural injection of CTB-SAP (or unconjugated CTB in controls animals) for retrograde labeling of phrenic motor neurons (Mantilla et al., 2009). We estimate a similar (but likely shorter) time frame for XII MN labeling following CTB-SAP (or unconjugated CTB) injection into the tongue. Moreover, pilot data from a few rats at Day 4 showed that XII degeneration has already started, yet VFSS and lickometer testing at Day 4 did not reveal any significant changes in licking/swallowing function. This collective preliminary data suggest the underlying XII degenerative process at Day 4 precedes the clinical onset of behavioral licking/swallowing deficits, as previously reported in the MND literature (Kiernan et al., 2011; Rattray et al., 2017; Salvadores et al., 2017). Thus, by starting the exercise program on Day 4, we were specifically attempting to prevent clinical onset and progression of dysphagia while also beneficially altering the underlying degenerative disease process in this model.

### Tongue Exercise Has a Beneficial Treatment Effect on Tongue Motility and Strength but Not Swallow Timing Measures

Here, we hypothesized that tongue exercise would preserve hypoglossal-tongue axis function and strength in CTB-SAP rats. This hypothesis held true for lick rate (i.e., tongue motility) and lick force (i.e., tongue strength), but not for swallow timing measures. Specifically, the swallow rate and lick-swallow ratio deficits identified in sham exercise-treated CTB-SAP rats were not any different from the exercised CTB-SAP rats; thus, there was no beneficial or harmful effect of tongue exercise on VFSS-derived swallow timing measures. These results perhaps can be attributed to lick versus swallow pattern generators (CGSs) in the brainstem (Jean, 2001; Boughter et al., 2007; Moore et al., 2014). Licking mainly involves the muscles of the tongue which are innervated by CN XII (hypoglossal) and the muscles of the jaw which are innervated by CN V (trigeminal), whereas the act of swallowing is more complex and extends beyond CNs V and XII to include CN VII (facial), CN IX (glossopharyngeal), CN X (vagus), CN XI (spinal accessory), and cervical spinal nerves. While the selective hypoglossal motor neuron degeneration in our rat model negatively impacts both lick and swallow CPGs, tongue exercise appears to have a beneficial effect mainly on the relatively simplistic CPG for licking. This suggests that our tongue exercise paradigm is highly specific for targeting the lick CPG but not the swallow CPG, even though rats were swallowing while performing “forceful licks” against the resisto-spout throughout the overnight exercise session. While this finding may simply be a limitation of behavioral research with rats (and laboratory animals in general), this species nonetheless provides an invaluable tool for improving the scientific understanding of normal and disordered swallowing to accelerate the advancement of personalized dysphagia treatment.

### Translational Methodology for Advancing Personalized Dysphagia Treatment

In this study, we used our rat model of dysphagia to develop translational methodology for a personalized strength endurance tongue exercise to ultimately benefit patients with MNDs. Specifically, rats performed an estimated 320 forceful licks against the exercise spout (i.e., resisto-spout) for fluid access per night of exercise. Importantly, the exercise program took place in each rat’s home cage, without water restriction, in an attempt to mimic personalized home exercise programs traditionally utilized for dysphagia rehabilitation. The resisto-spout force requirement during exercise was based on each individual rat’s MVLF at baseline force-lickometer testing. Thus, our approach resembled the dysphagia rehabilitation literature in which each patient’s baseline tongue force is measured by compressing a pneumatic pressure sensor between the tongue and hard palate, which is then used to develop a personalized tongue strengthening program (Lazarus et al., 2003, 2014; Robbins et al., 2005; Lazarus, 2006; Yeates et al., 2008; Steele et al., 2013; Park et al., 2015). Importantly, these prior clinical studies used a low-repetition/high-resistance exercise paradigm with long rest intervals (Lazarus et al., 2003, 2014; Robbins et al., 2005; Lazarus, 2006; Yeates et al., 2008) that resembles a body builder’s workout to increase muscle mass (Mangine et al., 2015).

A low-repetition/high-resistance tongue exercise paradigm has also been tested in a variety of rat models (e.g., ALS, primary aging, and Parkinson’s disease). The approach requires an extensive behavioral conditioning program (via water restriction) to train rats to press the tongue progressively harder against a force transducer to receive water rewards, typically 50–80% of maximum lick force for ∼20 “tongue presses” during brief (<10-min) treatment sessions, 3–5 days/week (Smittkamp et al., 2010; Ciucci et al., 2011, 2013; Kletzien et al., 2013; Cullins et al., 2018; Krekeler et al., 2018). Though beneficial effects were shown for rat models of primary aging (Kletzien et al., 2013; Cullins et al., 2018; Krekeler et al., 2018) and Parkinson’s disease (Ciucci et al., 2011, 2013), this tongue exercise training paradigm in the ALS rat model was detrimental, causing further reduction in lick rate during drinking and no discernable improvement in tongue strength (Ma et al., 2017). For this reason, we chose to investigate a high-repetition/low-resistance (∼40% greater than MVLF) exercise paradigm designed for strength endurance and flexibility (Anderson and Kearney, 1982) that may be more tailored to prevent the characteristic weakened, slowed, fatigued, and limited tongue motion caused by MNDs. Our results thus far show beneficial treatment effects in our rat model, without any harmful outcomes. Moreover, the exercise intensity level was sufficiently low that it did not produce any beneficial effects in control animals (i.e., ‘control’ + ‘sham exercise group’), thus providing evidence that this high-repetition/low-resistance training paradigm is uniquely tailored for degenerating motor neurons. However, given the degenerative underpinnings of our model as well as motor neuron diseases in general, frequent re-evaluation and adjustment of the tongue exercise intensity benchmark will likely be essential to optimize personalized treatment outcomes.

### Magnetic Resonance Imaging Detection of Degenerative Changes in the Hypoglossal-Tongue Axis

Here, we hypothesized that structural degeneration in the brainstem and tongue would be detectable with *in vivo* MRI and present in sham exercise-treated CTB-SAP rats based on our previous findings (i.e., hypoglossal motor neuron degeneration and genioglossal myofiber atrophy; Lind et al., 2021), and would be prevented or decreased with tongue exercise. While our observations thus far are based on a small subset of rats and treatment groups, MRI did provide evidence of degeneration in the hypoglossal-tongue axis (i.e., a trend for 4th ventricle enlargement, significantly increased tongue volume and thickness, and marked hyperintensity of the tongue) in response to genioglossal myofiber atrophy (Lind et al., 2021), and these pathological MRI features were somewhat mitigated via tongue exercise in CTB-SAP rats. Importantly, fourth ventricle enlargement is a common feature of neurodegenerative diseases and is correlated with degeneration of the surrounding brain structures (Westeneng et al., 2015; Bede et al., 2019). In the case of ALS, 4th ventricle enlargement is attributed to atrophy of the floor of the fourth ventricle due to degeneration of the hypoglossal and vagal trigones, which are formed by the underlying hypoglossal and vagal nuclei, respectively (Westeneng et al., 2015; Bede et al., 2019). In our CTB-SAP model, only the hypoglossal nucleus undergoes neurodegeneration (Lind et al., 2018); thus, it is intuitive to speculate that the observed 4th ventricle enlargement is solely due to degeneration of the hypoglossal trigone along the floor of the 4th ventricle. However, we will need to conduct MRI and corresponding histological experiments with larger group sample sizes to specifically test this hypothesis. Moreover, our MRI findings of diffuse hyperintensity and increased volume and thickness of the tongue are indicative of muscle fiber inflammation and fat infiltrations, which are consistent with denervation atrophy as a consequence of hypoglossal motor neuron degeneration (Alves, 2010; Borges, 2010). These pathological tongue-based MRI findings in our CTB-SAP model remarkably resemble macroglossia in ALS patients (McKee et al., 2013; Hensiek et al., 2020). Thus, our model may be particularly suited for studying pathological muscle changes that remain poorly understood in ALS and other neuromuscular disorders (Theret et al., 2021).

The ability of MRI to detect early degenerative changes in the entire XII axis (brainstem and tongue) of our rat model suggests early MRI detection may likewise be possible in patients with MNDs. Our findings therefore suggest that this non-invasive imaging modality may have untapped clinical potential to facilitate early differential diagnosis of MNDs from other neurological disorders, thus providing opportunity for earlier intervention and objective treatment monitoring when effective treatments become available. Because of its non-invasive nature and wide availability in hospitals, MRI of the tongue and brainstem is readily translatable to future MND clinical trials.

### Limitations

Several limitations must be considered when interpreting the results of this study. First, we used overnight water consumption as an indirect measure of lick frequency (i.e., exercise repetitions). We are currently working on incorporating home-cage contact lickometer technology to permit direct measurement of lick frequency for individual rats. Second, we calculated each rat’s MVLF during spontaneous drinking in our force-lickometer system, which does not provide a lick-force challenge during testing. In other words, we cannot incrementally increase the force setting to identify each rat’s maximum tongue force capacity, which is a capability of other systems (Ciucci et al., 2011, 2013; Cullins and Connor, 2019). As a solution, our future research with this model will include tongue force strain gauge measurements (Nagai et al., 2008; Ciucci et al., 2013) at the study endpoint (Day 9) to determine each rat’s maximum lick force for correlation with behavioral findings. Third, the exercise intensity in our study was not strictly controlled between rats, as it ranged from 20 to 53%. This was due to some rats not tolerating the 50% > MVLF setting, as evidence by switching to biting rather than licking behavior. For these cases, we reduced the resisto-spout force setting until rats resumed drinking. To overcome this limitation in future studies, we have been conducting pilot testing using a built-in “exercise force ramp up” period at the start of each overnight treatment, whereby the force setting is increased from 20% to 50% > MVLF in 10% increments every 15–30 min during the first 1.5 h. Our preliminary results suggest this approach prevents conversion to biting behaviors at higher force settings. Thus, we will use this approach to reduce the variability in exercise intensity in our future studies. Fourth, we were surprised to find that tongue exercise did not preserve swallow rate or lick-swallow ratio in CTB-SAP rats. The persistent slower swallow rate and higher lick-swallow ratios suggest that CTB-SAP rats take longer to accumulate a sufficiently large bolus in the vallecular space to trigger the pharyngeal swallow reflex. While we did not measure bolus size, we could certainly include this VFSS-based outcome measure (Lever et al., 2015a,b; Osman et al., 2020) in our future studies with this model to determine if tongue exercise in CTB-SAP rats results in larger, more normal-sized boluses. Fifth, we did not identify PTT deficits in CTB-SAP rats, which we suspect is a limitation of fluoroscopy frame rate (i.e., 30 fps) in rodents (Lever et al., 2015a,b; Osman et al., 2020). Although not significant, the sham exercise-treated CTB-SAP rats had the longest PTT, and the PTT distribution in exercised CTB-SAP rats was similar to the two control groups, suggesting a potential beneficial effect of tongue exercise on this swallow timing measure. Therefore, we are currently investigating if 60 fps imaging capability may unmask significant PTT deficits in this model to provide another translational outcome measure to objectively quantify treatment response. Finally, we have thus far focused on drinking-related measures of tongue and swallowing function. The tongue is also essential for mastication (Palmer et al., 1992); thus, we expect our rat model of XII LMN degeneration will also develop deficits in mastication and dyscoordination between mastication and swallowing. We will therefore include mastication-based VFSS measures in our future studies with this model.

### Significance

This study provides novel evidence in support of tongue exercise as a treatment for dysphagia in MNDs, which currently remains highly controversial in the absence of high rigor investigations (Dworkin and Hartman, 1979; Watts and Vanryckeghem, 2001; Plowman, 2015; Ma et al., 2017). Thus, we will continue utilizing our rat model to optimize tongue exercise dosing parameters and investigate corresponding treatment mechanisms of action for future translation to MND clinical trials. The next step is to leverage our rat model of XII LMN degeneration to optimize dosing parameters that are translatable to humans. While we started this scientific exploration using a 12-h exercise program in rats, we fully acknowledge that translation to humans will require much shorter treatment durations. Thus, our rat model will provide a suitable platform for high throughput investigations of exercise dosing parameters as well as investigations of treatment mechanisms of action for future translation to MND clinical trials. Moreover, we will utilize this model to determine whether targeted tongue exercise is directly impacting the dying versus surviving XII motor neurons, or a combination thereof, which may lead to the discovery of mechanistic targets to effectively delay, slow, or even reverse XII motor neuron degeneration to achieve more impactful clinical outcomes. As such, this research may have scalability to other cranial and/or spinal nerves affected by motor neuron diseases.

## Data Availability Statement

The raw data supporting the conclusions of this article will be made available by the authors, without undue reservation.

## Ethics Statement

The animal study was reviewed and approved by University of Missouri Animal Care and Use Committee.

## Author Contributions

NN, TL, CS, and AK worked on developing and maintaining the model. NN and TL designed the study. EM, RT, KO, NN, and TL organized and maintained the study databases. TL and NN prepared and performed all intralingual injections. EM, RT, KO, and CH performed the behavioral conditioning. EM, RT, KO, and TL performed the videofluoroscopic swallow study (VFSS) testing. EM, RT, MB, CH, AK, and TL analyzed VFSS videos. AH, FB, and TL validated the JawTrack™ software data. EM, RT, and TL performed the force-lickometer testing, analyzed the data, and calculated maximum voluntary lick force (MVLF) percentages. EM, RT, KO, AK, and TL performed overnight exercise. LM, KW, LL, NN, TL, CS, and AK performed the MRI. MG and TL performed the statistical analysis. TL, EM, and NN wrote the initial draft of the manuscript. TL prepared Figures 1–7 and corresponding figure legends. LM, KW, LL, NN, MG, and TL prepared Figures 8, 9 and corresponding legends. TL and RT prepared the Supplementary Videos. All authors reviewed and approved the manuscript.

## Conflict of Interest

The authors declare that the research was conducted in the absence of any commercial or financial relationships that could be construed as a potential conflict of interest.

## Publisher’s Note

All claims expressed in this article are solely those of the authors and do not necessarily represent those of their affiliated organizations, or those of the publisher, the editors and the reviewers. Any product that may be evaluated in this article, or claim that may be made by its manufacturer, is not guaranteed or endorsed by the publisher.
